# Association between Insulin Monotherapy versus Insulin plus Metformin and the Risk of All-Cause Mortality and Other Serious Outcomes: A Retrospective Cohort Study

**DOI:** 10.1371/journal.pone.0153594

**Published:** 2016-05-06

**Authors:** Sarah E. Holden, Sara Jenkins-Jones, Craig J. Currie

**Affiliations:** 1 The Institute of Primary Care and Public Health, School of Medicine, Cardiff University, Cardiff, United Kingdom; 2 Global Epidemiology, Pharmatelligence, Cardiff, United Kingdom; Kagoshima University Graduate School of Medical and Dental Sciences, JAPAN

## Abstract

**Aims:**

To determine if concomitant metformin reduced the risk of death, major adverse cardiac events (MACE), and cancer in people with type 2 diabetes treated with insulin.

**Methods:**

For this retrospective cohort study, people with type 2 diabetes who progressed to insulin with or without metformin from 2000 onwards were identified from the UK Clinical Practice Research Datalink (≈7% sample of the UK population). The risks of all-cause mortality, MACE and incident cancer were evaluated using multivariable Cox models comparing insulin monotherapy with insulin plus metformin. We accounted for insulin dose.

**Results:**

12,020 subjects treated with insulin were identified, including 6,484 treated with monotherapy. There were 1,486 deaths, 579 MACE (excluding those with a history of large vessel disease), and 680 cancer events (excluding those in patients with a history of cancer). Corresponding event rates were 41.5 (95% CI 39.4–43.6) deaths, 20.8 (19.2–22.5) MACE, and 21.6 (20.0–23.3) cancer events per 1,000 person-years. The adjusted hazard ratios (aHRs) for people prescribed insulin plus metformin versus insulin monotherapy were 0.60 (95% CI 0.52–0.68) for all-cause mortality, 0.75 (0.62–0.91) for MACE, and 0.96 (0.80–1.15) for cancer. For patients who were propensity-score matched, the corresponding aHRs for all-cause mortality and cancer were 0.62 (0.52–0.75) and 0.99 (0.78–1.26), respectively. For MACE, the aHR was 1.06 (0.75–1.49) prior to 1,275 days and 1.87 (1.22–2.86) after 1,275 days post-index.

**Conclusions:**

People with type 2 diabetes treated with insulin plus concomitant metformin had a reduced risk of death and MACE compared with people treated with insulin monotherapy. There was no statistically significant difference in the risk of cancer between people treated with insulin as monotherapy or in combination with metformin.

## Introduction

The rate of insulin use in type 2 diabetes increased more than six-fold in the UK between 1991 and 2010 [1]. In 1991, almost all patients using insulin for type 2 diabetes did so as monotherapy [1]. By 2010, 42% of these patients were prescribed insulin in combination with metformin; the percentage treated with insulin monotherapy decreased to 37% [1].

A position statement of the American Diabetes Association and the European Association for the Study of Diabetes recommends that metformin therapy be continued when insulin is initiated [2]. A Cochrane review reported that bedtime neutral protamine Hagedorn (NPH) insulin in combination with oral glucose-lowering therapies provided comparable glycaemic control to insulin monotherapy but with generally lower doses of insulin [3]. In addition to its potential for lowering concomitant insulin dose [3], metformin has been reported to have a number of benefits that might attenuate risk, including cardiovascular benefits [4–6], cancer-related benefits [7,8], and improved all-cause mortality [9]. In meta-analyses including observational studies, metformin has been shown to be associated with a decreased risk of cancer in observational studies [10–13]. However, these findings were not replicated in meta-analyses of RCT data [13,14].

We have previously reported a possible association between increasing insulin dose and increased all-cause mortality in people with type 2 diabetes treated with insulin monotherapy [15]. Previous data have also demonstrated a direct association between insulin exposure and mortality [16]. Roumie and colleagues have reported that the addition of insulin to existing metformin treatment was associated with an increased risk of cardiovascular events and all-cause mortality versus the addition of sulfonylurea in people with type 2 diabetes [17]. We recently reported that people with type 2 diabetes using insulin were at an increased risk of a combined endpoint defined as first major adverse cardiac event, first cancer, or mortality, with the risk being significantly higher for users of insulin monotherapy compared with users of insulin plus concomitant metformin [18]. A common criticism of retrospective observational studies is the risk of bias due to confounding by indication. To our knowledge, this is the first study to account for insulin exposure and to match the cohorts by propensity score.

The aim of this study was to determine whether combining insulin with metformin reduced the risk of all-cause mortality, major adverse cardiac event (MACE), or cancer compared with insulin monotherapy, taking insulin dose into account.

## Methods

### Data source

The data source was the Clinical Practice Research Datalink (CPRD) [19], a longitudinal database collating pseudonymized data collected in a non-interventional way from 660 participating primary-care practices throughout the UK. CPRD is representative of the UK population and contains 13 million patients, of which approximately five million are actively registered and can be followed prospectively. Data include demographics, medical history, investigations, and prescriptions. For a proportion of patients registered with a participating English practice, CPRD is linked to hospital data. Data were from January 2000 to January 2013. Patient consent is not required for the inclusion of their records in CPRD. Patients do however have the right to opt out if they do not want their records to be included in CPRD. Approval for this study was granted by the CPRD Independent Scientific Advisory Committee (reference number 11_017). The CPRD organization has obtained ethical approval from a National Research Ethics Service Committee (NRES) for all purely observational research using anonymized CPRD data; therefore, further ethical approval was not required.

### Patients

Firstly, we selected patients with type 2 diabetes. Patients were classified as having type 2 diabetes if they met at least one of the following criteria:

1. More than one explicit diagnosis of type 2 diabetes
2. Prescriptions for at least two differing classes of glucose-lowering medication other than insulin
3. At least one diagnosis of type 2 diabetes plus prescriptions for at least one glucose-lowering medication excluding insulin.

From this cohort of patients, we then selected those people who had been prescribed insulin. The study index date was defined as the date of first insulin prescription. It was required that the patient’s first recorded exposure to insulin should be in the form of insulin monotherapy or insulin in combination with metformin. For those patients prescribed insulin in combination with metformin, insulin was either added to pre-existing metformin therapy or metformin and insulin were started on the same date. Patients were required to have been registered at an up-to-standard practice for at least 365 days before the index date.

Patients were excluded if they had any record for secondary diabetes, a yearly average insulin dose greater than 4 units/kg/day, insulin prescribed on only one occasion, or no recorded weight. Patients with a prior history of large vessel disease (defined as myocardial infarction [MI], stroke, angina, or coronary revascularization) or cancer were excluded from analyses in which MACE or cancer were the respective endpoints. Only patients with at least two prescriptions for insulin were included because this was a requirement for the calculation of daily dose. Patients could receive the second prescription for insulin between 1 and 179 days following their first prescription for insulin (i.e. the index date) in order to be classed as still using insulin therapy. Because of this selection criterion, patients had to survive until they received their second prescription for insulin, but time to second prescription varied on a patient-by-patient basis. Therefore, in order to standardize this, we excluded all patients who were censored or had an event within 180 days of index (i.e. the maximum allowable time between insulin prescriptions before we assumed that their insulin regimen had been discontinued and restarted).

### Study endpoints

The primary outcome was all-cause mortality, with secondary endpoints of incident MACE (defined here as MI or stroke) and incident cancer (excluding non-melanoma skin cancer). Patients were followed from the index date plus 180 days to the earlier of the event (death, MACE, or cancer) date or the censor date. The censor date for all analyses was defined as the earliest of: the end of a patient’s recorded data, 90 days after their date of transfer to an alternative glucose-lowering regimen, or their last prescription for insulin. End of recorded data was defined as the earlier of the last data-collection date for the practice and the date a patient transferred to a different GP practice. An intention-to-treat analysis was also carried out, where patients were followed from the index date plus 180 days to the earlier of the event date or the end of the patient’s recorded data.

### Estimation of insulin dose

Weight-standardized daily insulin exposure (units/kg/day) was estimated from the volume of insulin prescribed. The methods have been detailed previously [15]. Briefly, for each insulin prescription, the quantity was converted to the number of international units prescribed and divided by the nearest recorded weight measurement. Cumulative average insulin dose was calculated on an annualized basis.

### Statistical methods

Kaplan–Meier curves stratified by treatment arm were produced for each endpoint. Time to each endpoint was evaluated using Cox proportional hazards modelling. Time zero for the Cox model and the calculation of the crude event rates was taken as the first prescription for insulin plus 180 days. People with events (cancer or MACE) prior to this date were excluded from the relevant analysis. The following baseline characteristics were included in the Cox proportional hazards model as continuous covariates: age, serum creatinine, body mass index (BMI), duration of diabetes, index year, Charlson comorbidity index [20], and the number of GP contacts in the year prior to index date. In order to account for the rapidly increasing risk of experiencing an adverse event as a person reaches old age, age was also added to the model as a quadratic term (in addition to the linear term) where significant (p<0.05). Metformin exposure, haemoglobin A1c (HbA1c), gender, insulin regimen, history of cancer or large vessel disease, smoking status, and prior exposure to lipid-lowering, anti-platelet, and anti-hypertensive therapies were included as categorical variables. For BMI, HbA1c, and serum creatinine, the baseline value was defined as the nearest record to the index date provided it was no more than 365 days before or 30 days after the index date. The search was conducted in the following order: −30, +30 and −365 days. For the Charlson index, history of large vessel disease and cancer, and prior exposure to-lipid-lowering, anti-platelet, and anti-hypertensive therapy, the patient’s record was searched for any relevant diagnoses or prescriptions recorded prior to the index date. Diabetes duration was calculated as the time between the diabetes presentation date (defined as the earlier of the first recorded diabetes diagnosis and the first prescription for a glucose-lowering therapy) and the index date. Smoking status at baseline was defined as the nearest recorded status prior to the index date. Insulin regimen was defined as the regimen (basal, basal–bolus, premix) prescribed to the patient during study follow-up. A fourth group, called ‘other’, comprised those patients who could not be categorized as basal, basal–bolus, or premix or who switched between regimen types. 15.7%, 12.3%, and 16.0% of patients had no BMI, HbA1c, or serum creatinine measurement at baseline, respectively. Missing baseline values were imputed using multiple imputation for BMI, HbA1c, and serum creatinine.

In the primary analysis, cumulative weight-standardized insulin exposure was estimated for each year following insulin initiation and analyzed as a time-dependent variable [16]. Other measures of insulin exposure were evaluated in sensitivity analyses. For the intention-to-treat analysis, mean insulin dose in year 1 was added to the Cox model.

Adjusted hazard ratios (aHRs) were calculated with 95% confidence intervals. The proportional hazards assumption was tested by examining the Pearson correlation between Schoenfeld residuals and the rank of survival time for cases that had progressed to death and also using Cox adjusted log–log curves. Where appropriate, interactions with time were used to assess the proportional hazards assumption. When the proportional hazards assumption was violated, Heaviside functions or interactions with time were used. Crude event rates were compared using tests of independence, where two-sided p-values were calculated using the mid-p exact test. Baseline characteristics were compared using the chi-squared test for categorical variables and t-test or Mann-Whitney U test for continuous variables, depending on their distribution. Levene’s test was employed to test for homogeneity of variances. If the assumption of equal variances was violated, a t test was conducted in which equal variances were not assumed.

Patients prescribed insulin monotherapy were matched to patients prescribed insulin plus metformin by propensity score, incorporating age, gender, year of index exposure, diabetes duration, BMI, serum creatinine, GP contacts in the year prior to index date, HbA1c, Charlson index, smoking status, history of prior cancer, history of prior large vessel disease, prior exposure to anti-hypertensive, lipid-lowering, or anti-platelet therapy, and line of therapy. Only patients with complete (i.e. non-imputed) data for the matching criteria were considered for matching. Propensity-score matching was carried out using IBM SPSS Statistics 20 using the SPSS R Essentials plug-in. Logistic regression was used to generate the propensity score. Nearest neighbour 1:1 matching was implemented and the calliper was set at 0.1 of the standard deviation of the logit of the propensity score. A series of checks was carried out to determine whether the covariates had been adequately balanced as a result of the matching criteria. These tests included an overall imbalance χ^2^ test developed by Hansen and Bowers, 2008; the relative multivariate imbalance L1 test by Iacus, King, and Porro, 2010; and the standardized mean difference for each covariate. Furthermore, diagnostic plots were examined to compare the distribution of propensity scores and the standardized difference for all terms before and after matching. The magnitude of the standardized differences for each covariate was also examined.

### Sensitivity analyses

In order to better identify the date of first diagnosis of type 2 diabetes and therefore the duration of diagnosed diabetes, sensitivity analyses were conducted. These analyses included only those patients who were registered at an up-to-standard practice for a minimum of 365 days prior to their diabetes presentation date. In a second sensitivity analysis, a subgroup analysis was carried out using quartiles of the number of different glucose-lowering regimens prescribed prior to the first prescription for insulin divided by the duration of diagnosed diabetes in order to account for differences in prescribed glucose-lowering therapy prior to the initiation of insulin. In all other respects, the specification of the Cox model remained as stated for the main analyses.

A further sensitivity analysis was conducted in order to further investigate the risk of all-cause mortality, MACE, and cancer across segmented portions of the follow-up period: 180–545 days post-index, 546–910 days post-index, 911–1,275 days post-index, 1,276–1,640 days post-index, and 1,641 days post index to end of follow-up. The Cox model was adjusted for the same covariates as in the primary analysis.

## Results

We identified 12,020 subjects with type 2 diabetes who progressed to insulin treatment, alone or in combination with metformin. Of these, 5,536 were prescribed insulin plus metformin and 6,484 were prescribed insulin monotherapy. Subjects were followed up for an average of 3.5 (median 2.5) years; a total follow-up of 41,747 patient-years.

### Baseline characteristics

Baseline characteristics at index date by regimen are detailed in Table 1, classifying each regimen as lower or higher insulin dose with respect to the median (0.648 units/kg/day). Patients treated with insulin plus metformin were younger than those treated with insulin monotherapy (median 61.0 years vs 67.0 years, p<0.001). People in the insulin plus metformin group had higher mean BMI (31.2kg/m^2^ vs 28.2kg/m^2^ for insulin plus metformin and insulin monotherapy, respectively, p<0.001) and lower median serum creatinine levels (83.0 vs 95.0 μmol/l, p<0.001). More patients receiving insulin monotherapy had a history of large vessel disease (27% vs 14% for insulin monotherapy and insulin plus metformin, respectively, p<0.001) and cancer (12% vs 8%, p<0.001). 2,757 patients prescribed insulin as monotherapy were matched to 2,757 patients prescribed insulin plus metformin by propensity score. The baseline characteristics for patients matched by propensity score are detailed in Table 2.

**Table 1:** Baseline characteristics by first exposure to each selected glucose-lowering regimen and average insulin dose^a^ over the study period. Lower-dose insulin: ≤0.648 units/kg/day; higher-dose insulin: >0.6480 units/kg/day (where the median insulin dose = 0.648 units/kg/day). DM, diabetes mellitus; BMI, body mass index; HbA1c, glycated haemoglobin; IQR, interquartile range; s.d., standard deviation.

Parameter	Insulin monotherapy						Insulin plus metformin						p-value^c^
	Lower dose		Higher dose		All		Lower dose		Higher dose		All		
Number of people, n (%)	3,087	(48%)	3,397	(52%)	6,484	(54%)	2,922	(53%)	2,614	(47%)	5,536	(46%)	
Males, n (%)	1,845	(60%)	1,798	(53%)	3,643	(56%)	1,682	(58%)	1,504	(58%)	3,186	(58%)	0.132
Age at index, years									59.5	(60.0)		.	
Mean (SD)	64.8	(14.7)	63.8	(14.0)	64.3	(14.3)	60.4	(12.4)	59.5	(11.2)	60.0	(11.9)	
Median (IQR)	67.0	(56.0–76.0)	66.0	(56.0–74.0)	67.0	(56.0–75.0)	62.0	(52.0–69.0)	60.0	(52.0–67.0)	61.0	(52.0–68.0)	<0.001
HbA1c, mean (SD), %	9.4	(2.2)	9.9	(2.2)	9.60	(2.2)	9.9	(1.9)	10.1	(1.7)	10.00	(1.8)	<0.001
Systolic blood pressure, mean (SD), mmHg	135.3	(19.6)	135.9	(19.5)	135.6	(19.5)	136.5	(17.3)	137.1	(17.1)	136.8	(17.2)	0.001
Smoking status													
Non-smoker, n (%)	1,156	(37%)	1,393	(41%)	2,549	(39%)	1,104	(38%)	987	(38%)	2,091	(38%)	
Ex-smoker, n (%)	1,303	(42%)	1,344	(40%)	2,647	(41%)	1,238	(42%)	1,133	(42%)	2,371	(43%)	
Current smoker, n (%)	628	(20%)	660	(19%)	1,288	(20%)	580	(20%)	494	(19%)	1,074	(19%)	0.080
Total cholesterol, mean (SD), mmol/l	4.7	(1.2)	4.7	(1.2)	4.7	(1.2)	4.6	(1.1)	4.6	(1.1)	4.60	(1.1)	<0.001
Index year, median (IQR)	2005	(2002–2008)	2004	(2002–2007)	2005	(2002–2008)	2006	(2004–2009)	2005	(2003–2008)	2006	(2003–2008)	<0.001
Serum creatinine, median (IQR), μmol/l	97	(77.0–132.0)	93	(76.0–123.0)	95.0	(77.0–128.0)	83	(71.0–98.0)	83	(71.0–97.0)	83.0	(71.0–98.0)	<0.001
DM duration, median (IQR), years	5.7	(1.8–10.7)	6.7	(2.7–11.5)	6.2	(2.2–11.1)	6.8	(3.3–10.8)	7	(4.1–11.1)	6.9	(3.7–10.9)	<0.001
BMI, mean (SD), kg/m^2^	28.4	(5.9)	28.1	(5.9)	28.2	(5.9)	31.1	(6.0)	31.4	(5.8)	31.2	(5.9)	<0.001
Prior glucose-lowering therapies, n (%)													
Acarbose	212	(7%)	284	(8%)	496	(8%)	212	(7%)	272	(10%)	484	(9%)	<0.001
DPP-4	97	(3%)	119	(4%)	216	(3%)	209	(7%)	140	(5%)	349	(6%)	<0.001
GLP-1	24	(1%)	46	(1%)	70	(1%)	124	(4%)	110	(4%)	234	(4%)	<0.001
Meglitinide	118	(4%)	151	(4%)	269	(4%)	146	(5%)	153	(6%)	299	(5%)	<0.001
Metformin	2,224	(72%)	2,676	(79%)	4,900	(76%)	2,736	(94%)	2,509	(96%)	5,245	(95%)	<0.001
SU	2,394	(78%)	2,978	(88%)	5,372	(83%)	2,261	(77%)	2,237	(86%)	4,498	(81%)	<0.001
TZD	786	(25%)	1,039	(31%)	1,825	(28%)	1,128	(39%)	1,011	(39%)	2,139	(39%)	<0.001
Other	4	(0%)	9	(0%)	13	(0%)	3	(0%)	4	(0%)	7	(0%)	0.015
General morbidity													
Prior large vessel disease, n (%)	887	(29%)	860	(25%)	1,747	(27%)	441	(15%)	353	(14%)	794	(14%)	<0.001
Prior cancer, n (%)	410	(13%)	374	(11%)	784	(12%)	234	(8%)	201	(8%)	435	(8%)	<0.001
Prior renal disease, n (%)	942	(31%)	940	(28%)	1,882	(29%)	527	(18%)	488	(19%)	1,015	(18%)	<0.001
Prior anti-hypertensives, n (%)	2,083	(67%)	2,377	(70%)	4,460	(69%)	2,027	(69%)	1,920	(73%)	3,947	(71%)	0.003
Prior lipid-lowering drugs, n (%)	1,744	(56%)	1,965	(58%)	3,709	(57%)	2,032	(70%)	1,876	(72%)	3,908	(71%)	<0.001
Prior anti-platelet drugs, n (%)	1,448	(47%)	1,576	(46%)	3,024	(47%)	1,387	(47%)	1,289	(49%)	2,676	(48%)	0.063
Charlson comorbidity index, median (IQR)	2	(1.0–4.0)	2	(1.0–4.0)	2.0	(1.0–4.0)	2	(1.0–3.0)	2	(1.0–3.0)	2.0	(1.0–3.0)	<0.001
GP contacts prior year, median (IQR)	15	(8.0–24.0)	15	(9.0–25.0)	15.0	(8.0–25.0)	13	(7.0–21.0)	13	(8.0–22.0)	13.0	(8.0–21.0)	<0.001
Insulin regimen^a^													
Basal–bolus	216	(7%)	432	(13%)	648	(10%)	264	(9%)	436	(17%)	700	(13%)	
Basal	436	(14%)	222	(7%)	658	(10%)	869	(30%)	361	(14%)	1,230	(22%)	
Premix	1,926	(62%)	1,794	(53%)	3,720	(57%)	1,372	(47%)	1,120	(43%)	2,492	(45%)	
Other or varies^b^	509	(16%)	949	(28%)	1,458	(22%)	417	(14%)	697	(27%)	1,114	(20%)	<0.001

^a^ During study follow-up.

^b^ This category also includes people who switched between the three different insulin regimens.

^c^ Comparison of all patients treated with insulin monotherapy versus all patients treated with insulin plus metformin. The methods for deriving all other baseline characteristics are detailed in the Statistical Methods section.

**Table 2:** Baseline characteristics for propensity-score matched patients.

Parameter	Insulin monotherapy		Insulin plus metformin		p-value
Number of people, n (%)	2,757	(50%)	2,757	(50%)	
Males, n (%)	1,584	(57%)	1,564	(57%)	0.586
Age at index, mean (median), years	61.9	(63.0)	61.8	(63.0)	0.066
HbA1c, mean (SD), %	9.9	(2.2)	9.9	(1.8)	0.776
Systolic blood pressure, mean (SD), mmHg	134.7	(18.4)	136	(17.5)	0.013
Smoking status:					0.084
Non-smoker, n (%)	1,043	(38%)	1,007	(37%)	
Ex-smoker, n (%)	1,153	(42%)	1,232	(45%)	
Current smoker, n (%)	561	(20%)	518	(19%)	
Total cholesterol, mean (SD), mmol/l	4.7	(1.2)	4.6	(1.1)	<0.001
Serum creatinine, median (IQR), μmol/l	86	(72.0–103.0)	87	(74.0–103.0)	0.062
DM duration, median (IQR), years	6.2	(2.6–11.0)	6.8	(3.6–10.9)	<0.001
BMI, mean (SD), kg/m^2^	29.2	(6.1)	29.7	(5.5)	
General morbidity:					
Prior large vessel disease, n (%)	524	(19%)	502	(18%)	0.446
Prior cancer, n (%)	285	(10%)	266	(10%)	0.394
Prior anti-hypertensives, n (%)	1,916	(69%)	1,932	(70%)	0.639
Prior lipid-lowering drugs, n (%)	1,874	(68%)	1,906	(69%)	0.353
Prior anti-platelet drugs, n (%)	1,303	(47%)	1,306	(47%)	0.936
Charlson comorbidity index, median (IQR)	2	(1.0–3.0)	2	(1.0–3.0)	0.515
GP contacts prior year, median (IQR)	15	(9–24)	14	(8–24)	0.130

### Crude event rates

**Fig 1:**
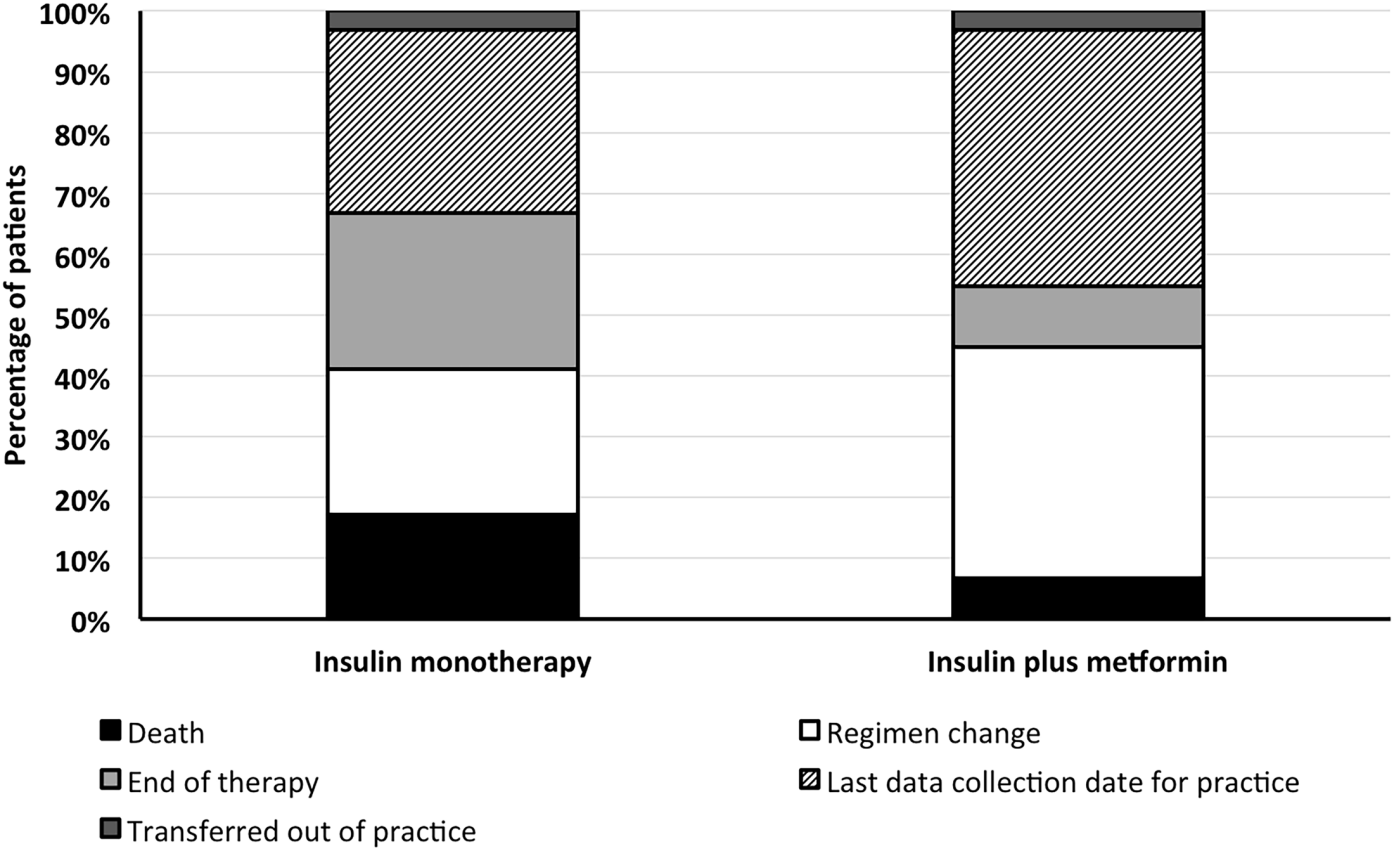
Reasons for censorship. Censorship crieria were applied in the order shown in the key below'.

Of those patients who survived until the end of follow-up, 36% of those prescribed insulin monotherapy and 45% of those prescribed insulin in combination with metformin were censored at their GP practice’s last data-collection date; 4% and 3%, respectively, were censored when they transferred out of practice. The percentage of patients censored by each of the criteria outlined in the methods is detailed in Fig 1.

There were 1,486 deaths amongst patients using any insulin regimen, a rate of 41.5 (95% CI 39.4–43.6) deaths per 1,000 person-years. After excluding people with prior MACE, 579 patients experienced MACE, a rate of 20.8 (19.2–22.5) events per 1,000 person-years. After excluding people with prior cancer, there were 680 cancer events, a crude event rate of 21.6 (20.0–23.3) cancer events per 1,000 person-years.

**Fig 2:**
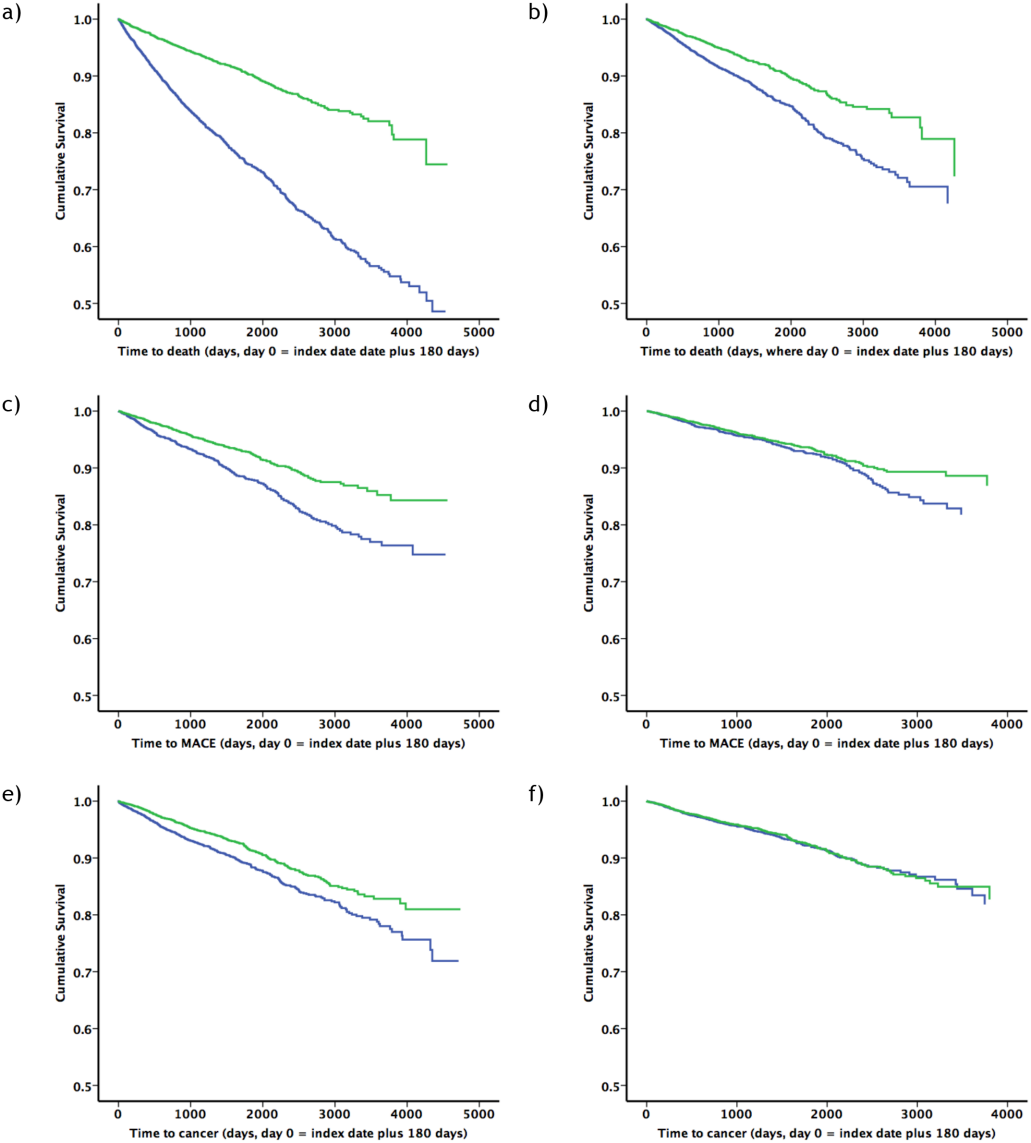
Kaplan–Meier and adjusted survival curves comparing insulin monotherapy and insulin plus metformin for all-cause mortality (a and b), MACE (c and d), and cancer (e and f). Blue = insulin monotherapy, green = insulin plus metformin. Time zero refers to index date plus 180 days. For the adjusted survival curves, a non-time-dependent Cox model was used, where insulin dose was modelled as the mean value for the follow-up period. The proportional hazards assumption was violated for history of cancer (all-cause mortality), history of receiving antihypertensive therapy (all-cause mortality), and serum creatinine (cancer).

Crude rates of all-cause mortality, MACE, and cancer were highest in people treated with insulin monotherapy: 61.3 versus 21.2 deaths per 1,000 person-years (p<0.001), 26.3 versus 15.9 MACE per 1,000 person-years (p<0.001), and 24.6 versus 18.7 cancer events per 1,000 person-years (p<0.001) for patients treated with insulin monotherapy versus insulin plus metformin, respectively (Table 3). Kaplan–Meier survival curves, stratified by treatment arm, are illustrated in Fig 2.

**Table 3:** Events, follow-up time, and crude event rates by glucose-lowering regimen and average insulin dose over the study period. Lower-dose insulin: ≤0.648 units/kg/day; higher-dose insulin: >0.6480 units/kg/day (where the median insulin dose = 0.648 units/kg/day).

	Insulin monotherapy			Insulin plus metformin		
	Overall	Low dose	High dose	Overall	Low dose	High dose
All-cause mortality						
Events	1,110	492	618	376	186	190
Patient-years	18,115	7,648	10,467	17,708	8,064	9,644
**Crude event rate (per 1,000 person-years)**	**61.3**	**64.3**	**59.0**	**21.2**	**23.1**	**19.7**
Cancer						
Events	382	165	217	298	137	161
Patient-years	15,553	6,495	9,058	15,936	7,309	8,626
**Crude event rate (per 1,000 person-years)**	**24.6**	**25.4**	**24.0**	**18.7**	**18.7**	**18.7**
MACE						
Events	342	132	210	237	109	128
Patient-years	12,983	5,392	7,591	14,860	6,741	8,118
**Crude event rate (per 1,000 person-years)**	**26.3**	**24.5**	**27.7**	**15.9**	**16.2**	**15.8**

Among the patients matched by propensity score, 534 out of 5,514 patients died, 235 out of 4,445 patients experienced an incident MACE, and 306 out of 4,909 patients experienced an incident cancer event. Overall, the event rates were 44.4 (95% CI 39.8–49.4) and 23.1 (20.1–26.4) deaths per 1,000 person-years; 22.2 (18.6–26.2) and 14.9 (12.3–18.0) MACE per 1,000 person-years; and 23.0 (19.5–26.9) and 19.9 (17.0–23.3) cancer events per 1,000 person-years for insulin monotherapy and insulin plus metformin, respectively.

### All-cause mortality

**Fig 3:**
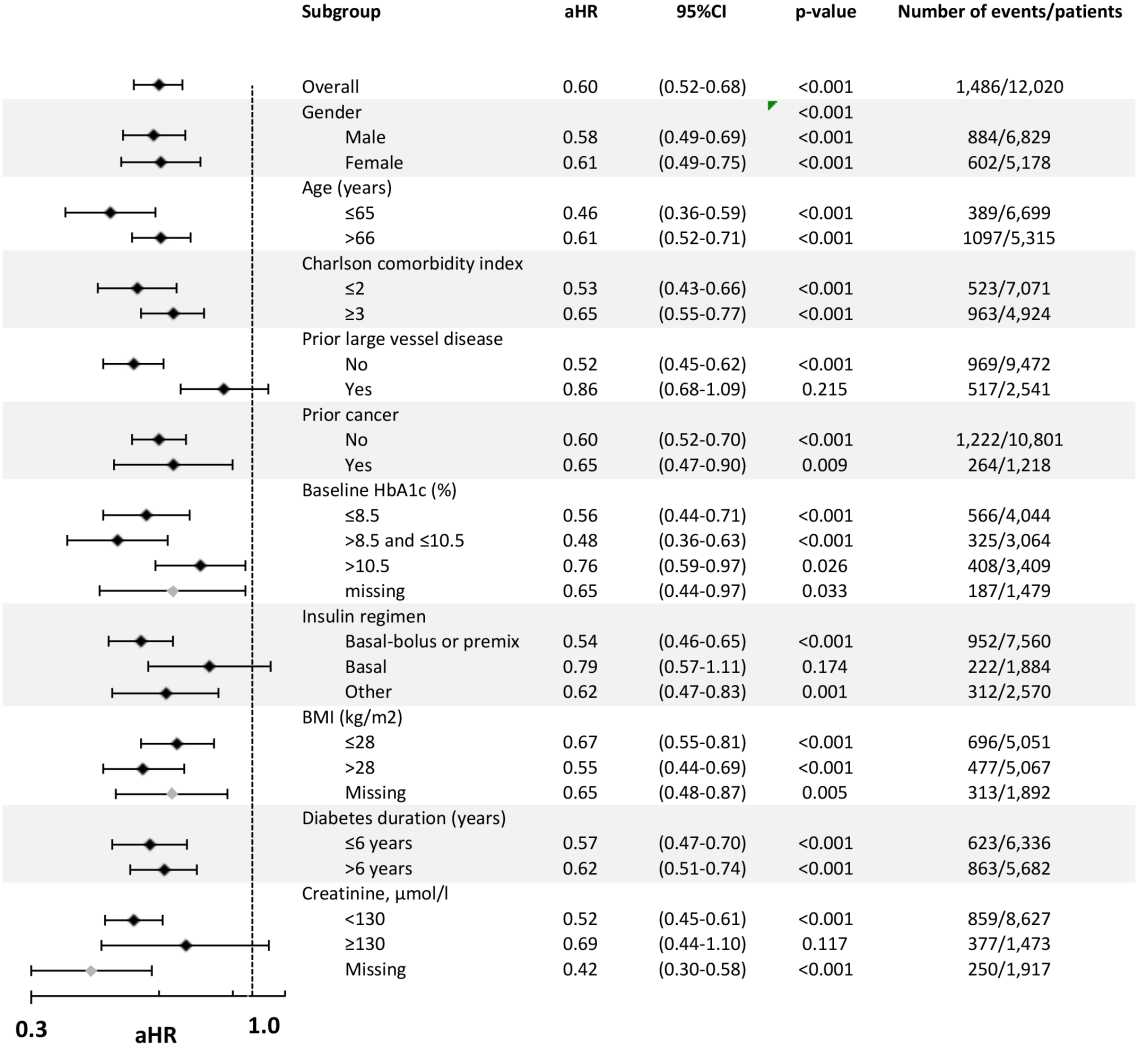
Adjusted hazard ratios for all-cause mortality for insulin plus metformin compared with insulin monotherapy. Notes: Final model specification: estimated cumulative insulin dose, therapy (±metformin), HbA1c, BMI, diabetes duration, index year, insulin regimen, smoking status, serum creatinine, prior cancer, prior large vessel disease, prior lipid-lowering therapy, prior anti-hypertensive therapy, prior anti-platelet therapy, prior GP contacts, Charlson comorbidity index, gender, and age at index. Insulin dose (units/kg/day) was added as a cumulative dose as an annually updated, time-dependent covariate. Baseline values were used for the remaining covariates as defined in the Statistical Methods section. Prior anti-hypertensive therapy and history of cancer violated the proportional hazards assumption of the Cox model and so were added as Heaviside functions (<1,095 and ≥1,095 days). The covariate used to categorize each subgroup was removed from the model for the respective analysis.

Across all insulin users, the aHR for all-cause mortality in relation to an increase in cumulative average insulin dose of 1 unit/kg/day was 1.48 (95% CI 1.30–1.70). The aHR for patients prescribed concomitant metformin—where patients with no exposure to metformin were used as the reference group—was 0.60 (0.52–0.68) (Fig 3). The aHRs for all covariates added to the Cox model are provided in Table 4. The point estimate for the aHR for patients prescribed concomitant metformin was lower than unity in all subgroups, but the confidence interval crossed unity in three of the 25 subgroups (Fig 3). The aHR for users of metformin was lower in people with an HbA1c >8.5% and ≤10.5% (0.48, 0.36–0.63), those aged ≤65 years (0.46, 0.36–0.59), those having less comorbidity (Charlson index of ≤2, 0.53, 0.43–0.66) or no prior large vessel disease (0.52, 0.45–0.62), and in those using basal–bolus or premix regimens (0.54, 0.46–0.65). A sensitivity analysis in which insulin exposure was estimated using a variety of methods prior to inclusion into the Cox model gave consistent results (Table 5).

**Table 4:** Adjusted hazard ratios for all-cause mortality for all covariates added to the Cox proportional hazards model. aHR: adjusted hazard ratio.

Covariates	aHR	95% CI	p-value
Time-dependent, annually updated cumulative insulin dose	1.48	(1.30–1.70)	<0.001
Glucose-lowering therapy			
Insulin monotherapy	1		
Insulin plus metformin	0.60	(0.52–0.69)	<0.001
Charlson comorbidity index	1.15	(1.11–1.20)	<0.001
BMI	1.00	(0.99–1.01)	0.639
Age	1.06	(1.05–1.07)	<0.001
Gender	0.88	(0.78–0.99)	0.027
GP contacts prior year	1.00	(1.00–1.01)	0.027
Smoking status			
Non-smoker	1		
Ex-smoker	1.17	(1.04–1.32)	0.009
Current smoker	1.63	(1.40–1.91)	<0.001
HbA1c			
Quintile 1	1		
Quintile 2	0.89	(0.76–1.04)	0.132
Quintile 3	0.92	(0.77–1.09)	0.315
Quintile 4	0.99	(0.83–1.19)	0.933
Quintile 5	1.12	(0.95–1.32)	0.194
Serum creatinine	1.00	(1.00–1.00)	0.001
Prior anti-hypertensive therapy			
<1,095 days	0.92	(0.77–1.09)	0.328
≥1,095 days	1.40	(1.10–1.77)	0.006
Prior anti-platelet therapy	1.06	(0.94–1.19)	0.331
Prior lipid-lowering therapy	0.74	(0.65–0.83)	<0.001
Prior large vessel disease	1.23	(1.08–1.40)	0.002
Prior cancer			
<1,095 days	1.48	(1.24–1.75)	<0.001
≥1,095 days	0.84	(0.63–1.13)	0.254
Duration of diagnosed diabetes	1.00	(1.00–1.00)	0.475
Insulin regimen			
Basal–bolus	1		
Basal	1.70	(1.27–2.29)	<0.001
Premix	1.42	(1.08–1.86)	0.011
Other or varies	0.98	(0.74–1.30)	0.895
Index year	0.98	(0.96–1.00)	0.037

**Table 5:** Sensitivity analysis exploring the effects of different estimations of insulin exposure on all-cause mortality in people prescribed insulin ± metformin.

Analytical approach to insulin dose	aHR for insulin ± metformin								
	All-cause mortality			MACE			Cancer		
Insulin dose covariate and model description	aHR	95%CI	p-value	aHR	95%CI	p-value	aHR	95%CI	p-value
Baseline continuous insulin dose^a^	0.6	(0.52–0.69)	<0.001	0.75	(0.62–0.91)	0.004	0.96	(0.80–1.15)	0.678
Continuous time-dependent covariate (cases with missing values excluded)^b^	0.62	(0.53–0.73)	<0.001	0.82	(0.65–1.04)	0.098	0.97	(0.79–1.20)	0.776
Continuous time-dependent covariate^b^	0.6	(0.52–0.68)	<0.001	0.75	(0.62–0.91)	0.004	0.96	(0.80–1.15)	0.639
Time-dependent insulin dose group (units/kg/day)^c^	0.59	(0.52–0.68)	<0.001						
Time-dependent insulin dose quartile (units/kg/day)^c^^d^	0.59	(0.52–0.68)	<0.001						
Time-dependent lag continuous insulin dose (year-1)^e^	0.59	(0.51–0.67)	<0.001	0.75	(0.62–0.91)	0.004	0.96	(0.80–1.15)	0.678
Time-dependent cumulative continuous insulin dose^f^	0.6	(0.52–0.68)	<0.001	0.75	(0.62–0.91)	0.004	0.96	(0.80–1.15)	0.668
Time-dependent continuous insulin dose (last year adjusted)^g^	0.59	(0.52–0.68)	<0.001	0.75	(0.61–0.91)	0.003	0.95	(0.80–1.14)	0.598
Time-dependent quartile of insulin dose (last year adjusted) ^d^^g^	0.63	(0.55–0.72)	<0.001						

^a^ Average daily, weight-standardized insulin dose in year 1, introduced into the Cox model as a continuous covariate.

^b^ Annually updated, average daily weight-standardized insulin dose introduced into the Cox model as a continuous covariate.

^c^ Annually updated, average daily weight-standardized insulin dose introduced into the Cox model as a categorical covariate.

^d^ Provided because there was evidence of a non-linear relationship between insulin dose and endpoint (tested by adding the squared dose as an annually updated covariate into the model in addition to the original annually updated insulin dose covariate and assessing if significant).

^e^ As b but a lag of one year applied.

^f^ Cumulative weight-standardized insulin exposure was estimated for each subsequent year following insulin initiation and analyzed as a time-dependent variable.

^g^ The dose for the final part year of follow-up was take as the average weight-standardized insulin dose for the 365-day period prior to the censor date for those patients with a follow-up of ≥365 days. Model specification: estimated insulin dose, therapy (±metformin), HbA1c, BMI, diabetes duration, index year, insulin regimen, smoking status, serum creatinine, prior lipid-lowering therapy, prior anti-hypertensive therapy, prior anti-platelet therapy, prior GP contacts, Charlson comorbidity index, gender, and age at index. Prior cancer was included in the Cox model for all-cause mortality and MACE endpoints. Prior large vessel disease was included for all-cause mortality and cancer endpoints. Age squared was included in the Cox model where cancer was the endpoint. Baseline values were used for all covariates other than insulin dose as defined in the Statistical Methods section. Prior anti-hypertensive therapy and history of cancer violated the proportional hazards assumption of the Cox model for all-cause mortality endpoint and so were added as Heaviside functions (<1,095 and ≥1,095 days). Regimen violated the proportional hazards assumption of the Cox model for the cancer endpoint and was therefore added as Heaviside functions (<1,095 and ≥1,095 days). For the MACE endpoint, interactions with time demonstrated that the proportional hazards assumptions was violated for insulin dose when it was added to the Cox proportional hazards model as an annually updated, time-dependent mean or cumulative mean value.

For patients matched by propensity score, the aHR for those prescribed concomitant metformin in comparison with those prescribed insulin monotherapy was 0.62 (95% CI 0.52–0.75).

For the intention-to-treat analysis, the aHR for all-cause mortality for patients prescribed insulin plus metformin when compared with insulin monotherapy was 0.70 (95% CI 0.64–0.77). The use of metformin violated the proportional hazards assumption of the Cox model. However, examination of the log–log curves revealed that the curves for insulin monotherapy and insulin plus metformin converged after 3,285 days but did not cross at any point.

### MACE

**Fig 4:**
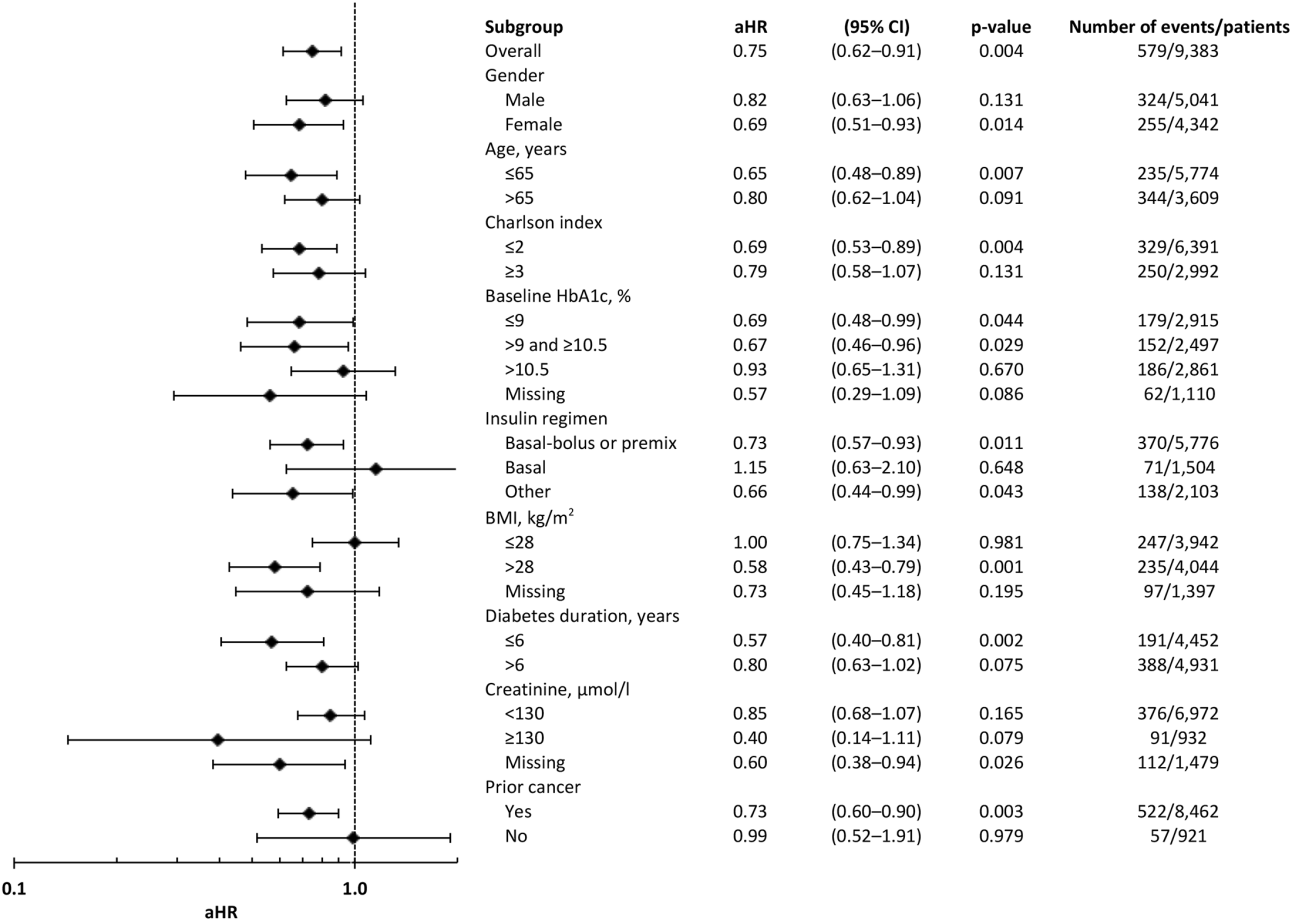
Adjusted hazard ratios for MACE for insulin plus metformin compared with insulin monotherapy. Notes: Final model specification: insulin exposure, therapy (±metformin), HbA1c, BMI, diabetes duration, index year, insulin regimen, smoking status, serum creatinine, prior cancer, prior lipid-lowering therapy, prior anti-hypertensive therapy, prior anti-platelet therapy, prior GP contacts, Charlson comorbidity index, gender, and age at index. Cumulative mean insulin dose (units/kg/day) was added as an annually updated, time-dependent covariate. Baseline values were used for the remaining covariates as defined in the Statistical Methods section.

There was no statistically significant association between annually updated, time-dependent cumulative mean insulin dose and MACE (aHR = 1.21, 95% CI 0.96–1.51, Fig 4). Patients treated with insulin plus concomitant metformin had a reduced risk of MACE (0.75, 0.62–0.91) when compared with those treated with insulin monotherapy. The aHRs for all covariates added to the Cox model are provided in Table 6. A sensitivity analysis in which insulin exposure was estimated using a variety of methods prior to inclusion into the Cox model gave consistent results (Table 5).

**Table 6:** Adjusted hazard ratios for MACE for all covariates added to the Cox proportional hazards model. Interactions with time demonstrated that the proportional hazards assumptions was violated for insulin dose.

Covariates	aHR	95% CI	p-value
Time-dependent, annually updated cumulative insulin dose	1.21	(0.96–1.51)	0.103
Glucose-lowering therapy			
Insulin monotherapy	1		
Insulin plus metformin	0.75	(0.62–0.91)	0.004
Charlson comorbidity index	1.18	(1.10–1.26)	<0.001
BMI	1.02	(1.00–1.03)	0.051
Age	1.04	(1.03–1.05)	<0.001
Gender	0.83	(0.69–1.00)	0.045
GP contacts prior year	1.01	(1.00–1.01)	0.138
Smoking status			
Non-smoker	1		
Ex-smoker	0.97	(0.80–1.18)	0.788
Current smoker	1.64	(1.31–2.06)	<0.001
HbA1c			
Quintile 1	1		
Quintile 2	0.88	(0.67–1.15)	0.340
Quintile 3	0.89	(0.68–1.18)	0.415
Quintile 4	1.12	(0.86–1.47)	0.393
Quintile 5	1.06	(0.80–1.42)	0.679
Serum creatinine	1.00	(1.00–1.00)	0.512
Prior anti-platelet therapy	1.34	(1.13–1.60)	0.001
Prior lipid-lowering therapy	1.10	(0.91–1.33)	0.328
Prior cancer	0.69	(0.51–0.94)	0.019
Duration of diagnosed diabetes	1.00	(1.00–1.00)	0.779
Insulin regimen			
Basal–bolus	1		
Basal	1.18	(0.77–1.79)	0.448
Premix	1.36	(0.95–1.95)	0.097
Other or varies	0.97	(0.66–1.41)	0.858
Index year	0.93	(0.89–0.96)	<0.001

The point estimate for the aHR for those prescribed insulin plus metformin when compared to those prescribed insulin as monotherapy was less than unity for many of the subgroups with the exception of those treated with basal insulin (aHR 1.15, 95% CI 0.63–2.10) and those with a BMI of ≤28kg/m^2^ (1.00, 0.75–1.34, Fig 4). Of the statistically significant results, the aHR for patients treated with concomitant metformin was lowest for those patients with a BMI of >28 kg/m^2^ (0.58, 0.43–0.79) and those with a duration of diagnosed diabetes of ≤6 years (0.57, 0.40–0.81).

For eligible propensity-score-matched patients, the use of metformin violated the proportional hazards assumption of the Cox model, therefore it was added as Heaviside functions. The aHR for those prescribed concomitant metformin compared with insulin monotherapy was 1.06 (95% CI 0.75–1.49) prior to 1,275 days and 1.87 (1.22–2.86) after 1,275 days.

For the intention-to-treat analysis, the aHR for patients prescribed insulin plus metformin when compared with insulin monotherapy was 0.86 (95%CI 0.74–1.00).

### Cancer

**Fig 5:**
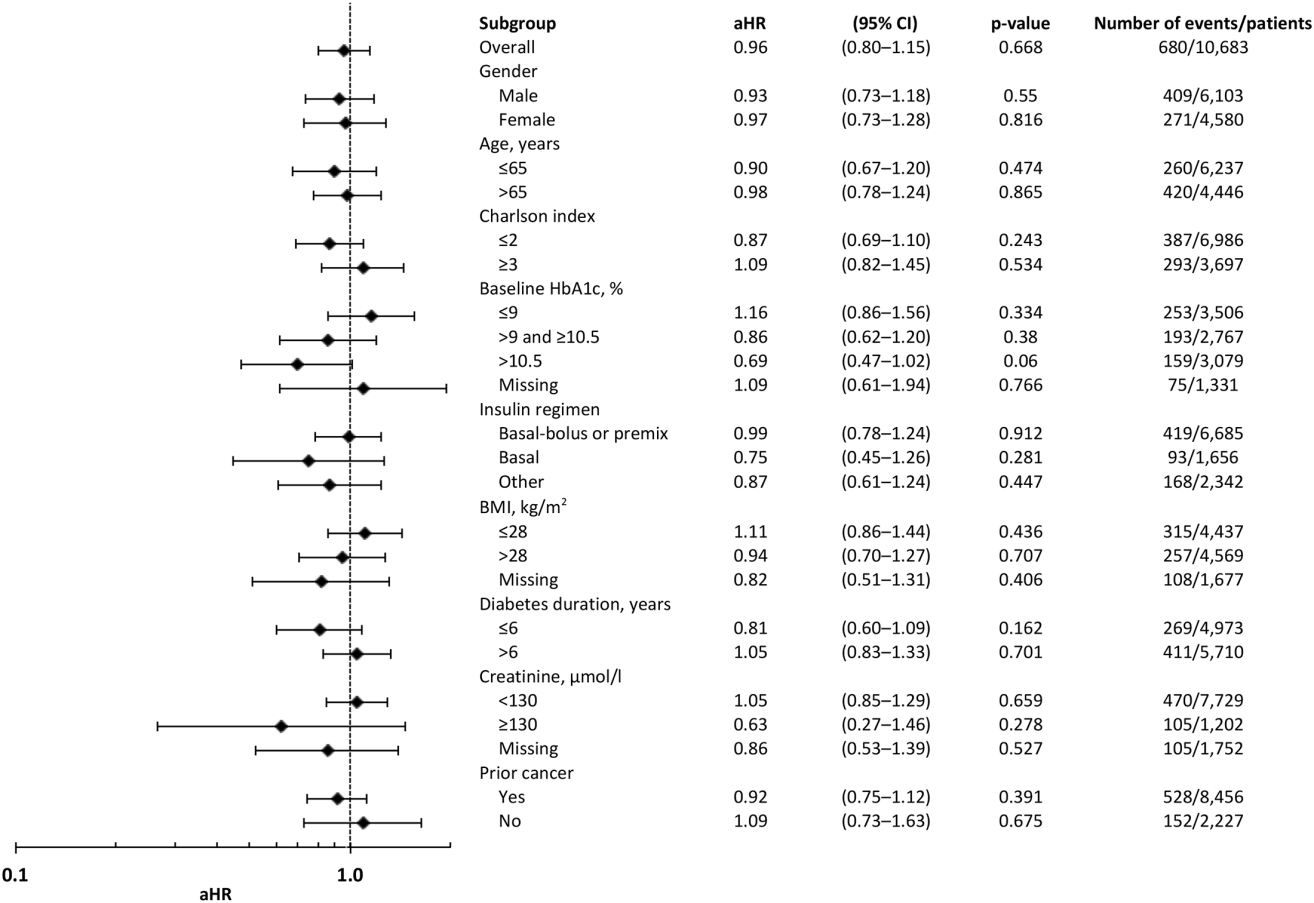
Adjusted hazard ratios for cancer for insulin plus metformin compared with insulin monotherapy. Notes: Final model specification: insulin exposure, therapy (±metformin), HbA1c, BMI, diabetes duration, index year, insulin regimen, smoking status, serum creatinine, prior large vessel disease, prior lipid-lowering therapy, prior anti-hypertensive therapy, prior anti-platelet therapy, prior GP contacts, Charlson comorbidity index, gender, and age at index. Insulin dose (units/kg/day) was added as a cumulative dose as an annually updated, time-dependent covariate. Baseline values were used for the remaining covariates as defined in the Statistical Methods section. Regimen violated the proportional hazards assumption of the Cox model and was therefore added as Heaviside functions (<1,095 and ≥1,095 days).

Across all insulin users, the aHR for cancer in relation to an increase in cumulative mean insulin dose of 1 unit/kg/day was 1.26 (95% CI 1.02–1.55, Fig 5). Patients treated with insulin plus concomitant metformin did not have a statistically significantly reduced risk of cancer (0.96, 0.80–1.15) when compared with those treated with insulin monotherapy. The aHRs for all covariates added to the Cox model are provided in Table 7. There was no statistically significant difference in the risk of cancer for those patients prescribed insulin in combination with metformin when compared with those prescribed insulin as monotherapy in any of the subgroups analyzed. Results of a sensitivity analysis in which insulin exposure was estimated using a variety of methods prior to inclusion into the Cox model are provided in Table 5.

**Table 7:** Adjusted hazard ratios for cancer for all covariates added to the Cox proportional hazards model.

Covariates	aHR	(95% CI)	p-value
Time-dependent, annually updated cumulative insulin dose	1.26	(1.02–1.55)	0.033
Glucose-lowering therapy	0.96	(0.80–1.15)	0.668
Insulin monotherapy			
Insulin plus metformin			
Charlson comorbidity index	1.12	(1.05–1.19)	<0.001
BMI	1.01	(0.99–1.02)	0.438
Age			
Linear term	1.22	(1.13–1.32)	<0.001
Quadratic term	1.00	(1.00–1.00)	<0.001
Gender	0.83	(0.70–0.98)	0.027
GP contacts prior year	1.00	(0.99–1.00)	0.578
Smoking status			
Non-smoker	1		
Ex-smoker	1.04	(0.87–1.24)	0.656
Current smoker	1.35	(1.08–1.68)	0.009
HbA1c			
Quintile 1	1		
Quintile 2	0.80	(0.61–1.05)	0.103
Quintile 3	0.80	(0.62–1.04)	0.094
Quintile 4	0.94	(0.72–1.24)	0.674
Quintile 5	0.82	(0.60–1.12)	0.211
Serum creatinine	1.00	(1.00–1.00)	0.462
Prior anti-hypertensive therapy	0.94	(0.77–1.14)	0.516
Prior anti-platelet therapy	1.06	(0.90–1.26)	0.471
Prior lipid-lowering therapy	0.90	(0.76–1.08)	0.270
Prior large vessel disease	0.77	(0.62–0.96)	0.017
Duration of diagnosed diabetes	1.00	(1.00–1.00)	0.009
Insulin regimen			
<1,095 days^a^			
Basal–bolus	1		
Basal	0.82	(0.52–1.31)	0.410
Premix	1.16	(0.84–1.61)	0.374
Other or varies	1.20	(0.94–1.52)	0.150
≥1,095 days^a^			
Basal–bolus	1		
Basal	1.52	(0.94–2.46)	0.090
Premix	1.35	(0.87–2.10)	0.178
Other or varies	1.30	(0.98–1.73)	0.073
Index year	1.01	(0.98–1.04)	0.468

^a^ Regimen violated the proportional hazards assumption of the Cox model for the cancer endpoint and was therefore added as Heaviside functions

For eligible propensity-score-matched patients, the aHR for those prescribed concomitant metformin compared with insulin monotherapy was 0.99 (95% CI 0.78–1.26).

For the intention-to-treat analysis, there was no statistically significant association between the use of metformin in combination with insulin and the cancer endpoint (aHR 0.977, 95% CI 0.84–1.13).

### Comparison of lower-dose and higher-dose insulin

**Fig 6:**
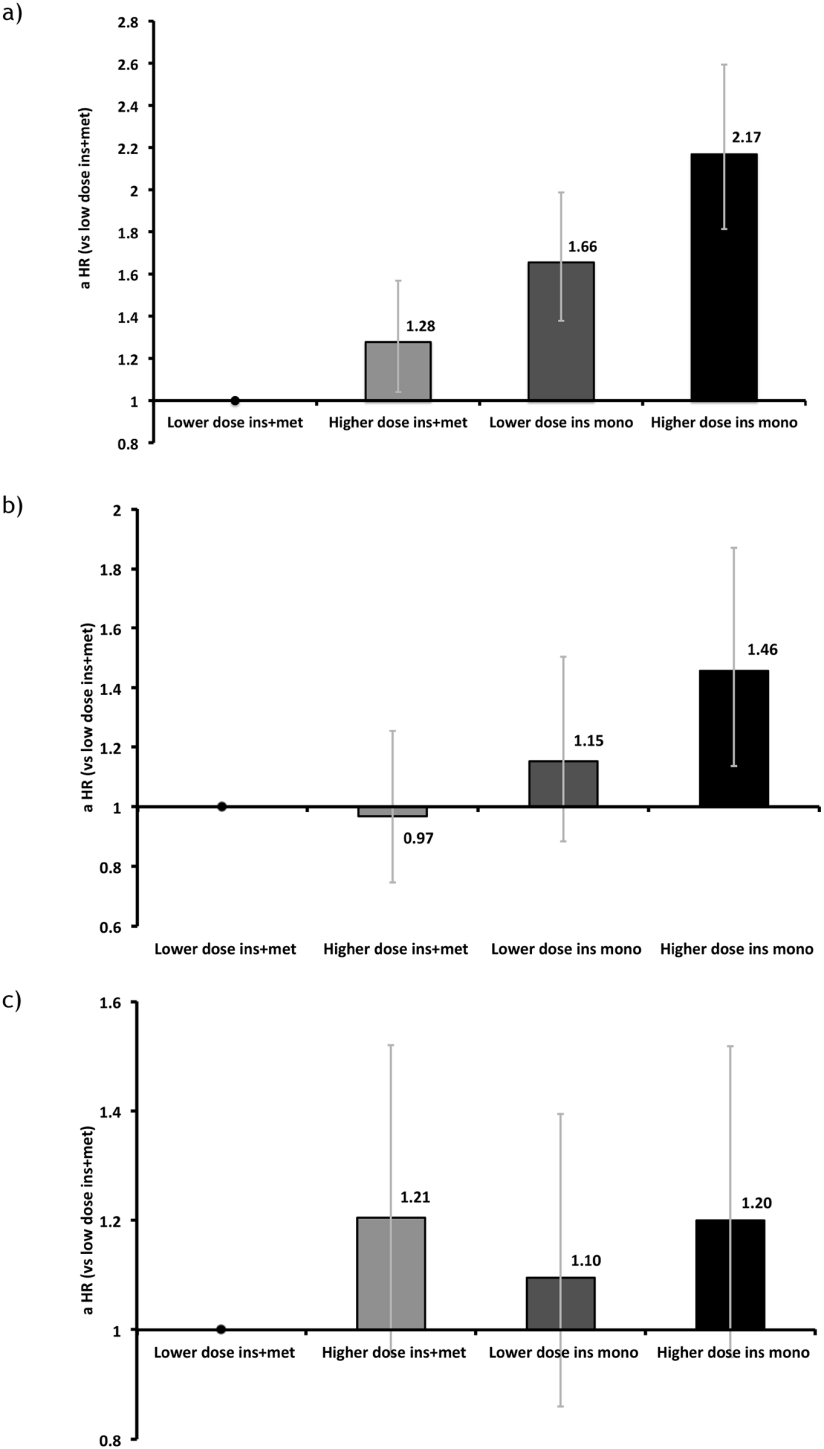
Adjusted hazard ratios for a) all-cause mortality, b) MACE, and c) cancer by lower and higher cumulative insulin dose. Notes: Model specification: categorical variable comprising insulin exposure and therapy (lower- and higher-dose insulin monotherapy, and lower- and higher-dose insulin plus metformin), HbA1c, BMI, diabetes duration, index year, smoking status, serum creatinine, prior cancer, prior large vessel disease, prior lipid-lowering therapy, prior anti-hypertensive therapy, prior anti-platelet therapy, prior GP contacts, Charlson comorbidity index, gender, insulin regimen, and age at index. The combined insulin exposure and therapy variable was added to the Cox model as an annually updated, time-dependent covariate. Baseline values were used for the remaining covariates as defined in the Statistical Methods section. Lower insulin dose was defined as ≤0.648 units/kg/day and higher dose was defined as >0.648 units/kg/day. For all-cause mortality, prior cancer and prior anti-hypertensives violated the proportional hazards assumption of the Cox model and so were entered as Heaviside functions (≤1,095 and >1,095 days). For cancer, insulin regimen type violated the proportional hazards assumption of the Cox model and so this was added as Heaviside functions (<1,095 and ≥1,095 days).

There was a significantly increased risk of all-cause mortality for patients in the higher-dose insulin plus metformin and lower- and higher-dose insulin monotherapy groups relative to lower-dose insulin plus metformin (aHR 1.28, 95% CI 1.04–1.57; 1.66, 1.38–1.99; 2.17, 1.81–2.59; respectively [Fig 6a]). The risk of MACE was not significantly different in those treated with lower- and higher-dose insulin in combination with metformin (Fig 6b). Those prescribed higher-dose insulin monotherapy had the highest risk of MACE (aHR 1.46, 95% CI 1.14–1.87, versus lower-dose insulin in combination with metformin). For the cancer endpoint, higher-dose insulin was associated with a higher risk of cancer; however, these results were not statistically significant (aHR 1.21, 95% CI 0.96–1.52, for those prescribed higher-dose insulin plus metformin, and 1.20, 0.95–1.52, for those prescribed higher-dose insulin monotherapy versus lower-dose insulin plus metformin, Fig 6c).

### Sensitivity analyses

#### Patients with a wash-in of ≥365 days prior to diabetes presentation

For those patients with a wash-in of at least 365 days to diabetes presentation from the later of their registration date and practice up-to-standard date, 410 and 145 deaths were observed for those treated with insulin monotherapy and insulin plus metformin, respectively. The crude death rates were 49.5 and 19.1 per 1,000 person-years, respectively (p<0.001). For MACE (after excluding people with a history of large vessel disease), 128 and 75 events were observed for people treated with insulin monotherapy and insulin plus metformin, respectively. The crude event rates were 20.7 and 11.7 events per 1,000 person-years, respectively (p<0.001). For cancer (after excluding people with a history of cancer), 158 and 121 events were observed for people treated with insulin monotherapy and insulin plus metformin, respectively. The crude event rates were 22.2 and 17.6 events per 1,000 person-years, respectively (p = 0.05).

The use of metformin in people prescribed insulin was associated with a reduced risk of all-cause mortality (aHR 0.64, 95% CI 0.52–0.80), and MACE (0.64, 0.45–0.91). However, there was no significant difference in the risk of cancer when those prescribed insulin plus metformin were compared with those prescribed insulin monotherapy (0.90, 0.67–1.19). The use of metformin in combination with insulin was associated with a reduced risk of all-cause mortality for each quartile of the number of prior glucose-lowering regimens prescribed per year of diagnosed diabetes, but the reduction was only statistically significant in quartiles 1 and 3 (Table 8). The reduction in the risk of MACE and cancer associated with use of metformin was not significant in any quartile. However, for MACE, the aHR for the use of metformin was lowest in those in the first quartile of the number of prior glucose-lowering regimens prescribed per year and nearly achieved statistical significance (aHR 0.46, 95% CI 0.21–1.03).

**Table 8:** Adjusted hazard ratios for all-cause mortality, MACE, and cancer for patients with > = 365 days wash-in to diabetes presentation from the later of registration date and practice up-to-standard date. For the overall model, there was evidence that the proportional hazards assumption was violated for insulin dose. Therefore, an interaction with time was incorporated into the Cox model. For all-cause mortality, Heaviside functions (<1,095 and > = 1,095 days) were used for history of cancer and antihypertensives. Where the analysis was split by quartile of the number of prior glucose-lowering regimens per year, Heaviside functions were used for HbA1c (<730 and > = 730 days), history of cancer, and antiplatelet therapy (<365 and > = 365 days) for the MACE endpoint. For the cancer endpoint, history of receiving prescriptions for lipid-lowering therapy, gender (<1,095 and > = 1,095 days), diabetes duration (<1,460 and > = 1,460 days), and history of receiving antihypertensives (<365 and > = 365 days) were introduced into the model as Heaviside functions.

	All-cause mortality			MACE			Cancer		
	aHR	(95% CI)	n/N	aHR	(95% CI)	n/N	aHR	(95% CI)	n/N
Overall	0.64	(0.52–0.80)	555/5,783	0.64	(0.45–0.91)	203/4,592	0.90	0.67–1.19	279/5,110
Quartile of number of prior glucose-lowering regimens per year									
1	0.60	(0.37–0.98)	128/1,427	0.46	(0.21–1.03)	48/994	0.86	(0.41–1.78)	50/1,231
2	0.68	(0.45–1.02)	162/1,427	0.66	(0.35–1.27)	59/1,125	0.82	(0.45–1.49)	66/1,235
3	0.59	(0.38–0.91)	144/1,429	0.85	(0.44–1.63)	60/1,161	0.96	(0.58–1.57)	94/1,286
4	0.75	(0.45–1.25)	120/1,427	0.52	(0.21–1.28)	35/1,257	0.97	(0.54–1.74)	68/1,289

#### Risk of all-cause mortality, MACE, and cancer for segmented periods of follow-up

The use of metformin in combination with insulin was associated with a reduced risk of all-cause mortality in all periods of follow-up. These reductions were statistically significant in all follow-up periods with the exception of 1,276–1,640 days post-index (Table 9). The use of metformin in combination with insulin reduced the risk of MACE in all follow-up periods with the exception of 911–1,275 days post-index. However, these reductions were only statistically significant in the 180–545 days post-index (aHR, 0.65, 95% CI 0.45–0.93) and from 1,640 post-index to end of follow-up (0.65, 0.45–0.96). No statistically significant reduction in the risk of cancer was observed in any of the segmented follow-up periods.

**Table 9:** Adjusted hazards ratios for all-cause mortality, MACE and cancer for 365 day periods of follow-up.

Time since index date (days)	All-cause mortality			MACE			Cancer		
	aHR (insulin plus metformin vs insulin monotherapy)	95%CI	Event rate	aHR (insulin plus metformin vs insulin monotherapy)	95%CI	Event rate	aHR (insulin plus metformin vs insulin monotherapy)	95%CI	Event rate
180–545	0.60^a^	(0.47–0.76)	460/12,020	0.65^c^	(0.45–0.93)	167/9,358	0.90	(0.65–1.25)	201/10,658
546–910	0.59^b^	(0.43–0.79)	309/8,154	0.82	(0.52–1.30)	105/6,361	0.86	(0.57–1.31)	132/7,239
911–1,275	0.60	(0.41–0.87)	202/6,068	1.29	(0.78–2.14)	86/4,742	1.16^a^	(0.69–1.95)	85/5,363
1,276–1,640	0.69^a^	(0.45–1.07)	142/4,633	0.68^d^	(0.37–1.26)	62/3,602	0.85	(0.48–1.52)	67/4,086
1,641–end	0.58	(0.44–0.76)	373/3,499	0.65	(0.45–0.96)	159/2,721	1.13	(0.80–1.59)	195/3,082

^a^ History of large vessel disease was added as Heaviside functions (< and > = 180 days).

^b^ History of receiving prescriptions for antihypertensives was added as Heaviside functions (< and > = 180 days).

^c^ Age at index date was added as Heaviside functions (< and > = 180 days).

^d^ Charlson index was added as Heaviside functions (< and > = 180 days).

## Discussion

The risk of all-cause mortality and MACE was reduced in insulin-treated people receiving concomitant metformin when compared to those prescribed insulin as monotherapy. For all-cause mortality, this finding was stable across a range of diabetes-related phenotypic subgroups and after propensity-score matching. However, no significant difference in the risk of cancer between the two treatment groups was observed. Importantly, we accounted for insulin dose.

An association between the concomitant use of metformin and serious adverse events in people prescribed insulin has been reported previously. The HOME (Hyperinsulinemia: the Outcome of its Metabolic Effects) trial found that the addition of metformin to insulin therapy reduced the risk of macrovascular disease (hazard ratio 0.61, 95% CI 0.40–0.94) [6]. In a study conducted using data from CPRD, insulin monotherapy was reported to be associated with a significantly higher risk of MACE, cancer, or death when compared with insulin in combination with metformin (1.51, 1.28–1.78) [18]. However, this study did not account for insulin dose. Evidence from the Danish National Patient Register found that the aHR for all-cause mortality in comparison with sulfonylurea therapy was 0.96 (0.82–1.13) for insulin plus metformin and 1.14 (1.06–1.20) for insulin monotherapy [21]. Conversely, a more recent systematic review of RCTs conducted by Hemmingsen and colleagues reported that insulin plus concomitant metformin was not associated with a reduced risk of all-cause or cardiovascular mortality compared with insulin alone in people with type 2 diabetes [22]. This meta-analysis was limited, however, by the small number of reported events of interest [22]. In the United Kingdom Prospective Study, metformin treatment was associated with a 33% (95% CI 13–47%) reduction in all-cause mortality when compared with conventional therapy (predominately diet alone) [23]. Clinical guidelines recommend that when insulin treatment is started it is added to, rather than replaces, existing metformin therapy [2]. Among its purported benefits, metformin may be cardioprotective, an effect that cannot be solely explained by its ability to lower blood glucose [4,5]. Systematic reviews have reported that insulin plus metformin cause improved glycaemic control, less weight gain, and reduced insulin requirements when compared with insulin monotherapy [3,22]. The REACH (Reduction of Atherothrombosis for Continued Health) study selected patients with atherothrombosis and showed that treatment with metformin was associated with an aHR for mortality of 0.76 (95% CI 0.65–0.89) compared with no metformin [24]. Metformin has been shown to attenuate the risk of all-cause mortality and other serious events when added to insulin [25].

*In vitro* studies suggest that metformin may protect against cancer through the activation of activated protein kinase (AMPK) [7]. However, we have not found a statistically significant difference in the risk of incident cancer between patients treated with insulin monotherapy or insulin in combination with metformin. In a retrospective observational study using data from the General Practice Research Database (GPRD, the predecessor of CPRD), van Staa and colleagues observed increased cancer risk for insulin versus metformin monotherapy in the first six months after initiation (adjusted relative rate 1.79, 95% CI 1.53–2.10), but this decreased with time from initiation, indicating protopathic bias [26]. Some meta-analyses of observational data have reported that metformin is associated with a decreased risk of cancer [10–13]. Conversely, Stevens and colleagues reported that, when data from RCTs were combined, metformin did not reduce the risk of cancer versus active comparators (0.98, 95% CI 0.77–1.23) [14].

The ORIGIN (Outcome Reduction with an Initial Glargine Intervention) trial [27] showed that, when compared with standard care, low-dose insulin glargine was not associated with a significant increase in the risk of cancer and cardiovascular outcomes. However, by the end of the study, 47% of the patients allocated to the insulin glargine group were also receiving metformin therapy (compared with 60% in the standard-care group) and 47% of patients in the standard-care group had received treatment with sulfonylureas and 11% insulin. Here we have found that, after adjusting for cumulative insulin exposure, people prescribed metformin in combination with insulin had a reduced risk of all-cause mortality in comparison with people prescribed insulin monotherapy, but no reduced risk of cancer was found. Associations between insulin dose and all-cause mortality have been reported previously [16,28]. Possible explanations for this dose association have been discussed [15].

There are several potential limitations to consider, some of which have been discussed previously [15]. Retrospective observational studies can only demonstrate possible associations with events; prospective randomized controlled trials are required to establish causality. Since these data are from routine practice, some data were missing. However, the data quality in CPRD is generally considered to be good [29], and only those patient records meeting CPRD’s research quality criteria were included. Rules were applied to maintain consistency in the selection of patients with type 2 diabetes. However, misclassification of diabetes type was possible and was more likely to affect patients prescribed insulin monotherapy. Duration of diagnosed type 2 diabetes was used as a covariate in the Cox model. In order to calculate this parameter in those patients who registered at the GP practice with pre-existing diabetes, we relied on the GP recording the date of first diagnosis rather than the date that the current GP practice became aware of the condition. As this may not be accurate in all cases, a sensitivity analysis was carried out using only those patients with an adequate wash-in between registration with the GP practice and first prescription for a glucose-lowering therapy. The risk of adverse events obtained from the sensitivity analyses was similar to those obtained from the main analyses.

There were potential limitations to the methods used to estimate insulin dose. Under- and overestimations of prescribed quantities were possible due to inconsistencies between fields in the prescriptions table or ambiguities in the quantities prescribed, but rules were devised to maintain consistency [15]. The exclusion of people with no weight measurement may have led to the elimination of sicker or very obese patients where weight measurement is more challenging. There is a theoretical time bias arising from the incorporation of the year 1 and the time-dependent, annually updated mean and cumulative mean insulin dose into the Cox model. However, similar aHRs for the use of metformin were obtained when insulin dose was introduced into the Cox model as an annually updated mean value with a lag of one year.

Insulin exposure was calculated from the volume of insulin prescribed. We were unable to determine from the data whether the patient collected or used all the insulin prescribed to them. A larger proportion of patients in the higher-dose insulin groups switched between basal–bolus, premix, or basal insulin regimens. At each switch, a certain amount of insulin is likely to be wasted, leading to an overestimation of their insulin dose. These patients are also likely to be the least well controlled.

One of the main criticisms of this type of observational study is the possibility of confounding by indication. This has been minimized here by our selecting only those patients initiated on insulin; however, differences in baseline characteristics did exist. For example, patients receiving insulin in combination with metformin were in generally heavier than those receiving insulin as monotherapy and less likely to have a history of large vessel disease. Due to the risk of lactic acidosis, metformin should be used with caution in renal impairment [30]. At baseline, 28% of patients in the insulin monotherapy group had a prior history of renal disease versus 18% in the insulin plus metformin group. In addition, 19% of patients in the insulin monotherapy group had a creatinine level of >130μmol/l in comparison with 3% in the insulin plus metformin group; this did not impact our findings in sensitivity analysis. Metformin should also be withdrawn in people at risk of tissue hypoxia or deterioration in renal function, such as those with acute heart failure or recent myocardial infarction, whereas there are no such barriers to receiving insulin as monotherapy. Therefore, the population of people receiving insulin in combination with metformin may be healthier than the monotherapy group. Measures of comorbidity were included in the Cox proportional hazards model and propensity-score matching, including the Charlson index; BMI; prior large vessel disease; history of prescriptions for anti-hypertensive, lipid-lowering and anti-platelet therapies; serum creatinine; and the number of GP contacts in the year prior. However, residual confounding may still exist. Censoring patients at time of regimen change may have also led to bias, where change in regimen could indicate poor glycaemic control. For people treated with insulin in combination with metformin, metformin therapy may be withdrawn in people developing contraindications to metformin therapy—for example, heart failure and renal impairment—leading to censorship. However, in our intention-to-treat analysis, the use of metformin in people prescribed insulin was still associated with a lower risk of all-cause mortality, and risk approached significance for the MACE endpoint (p = 0.051). There is an argument that increasing insulin dose could be a measure of diabetes deterioration. However, when, as a sensitivity analysis, insulin dose was entered into the Cox model as dose in year 1, the aHRs were 1.40 (1.22–1.60) for all-cause mortality and 1.22 (1.07–1.40) for the combined endpoint.

The number of events should be at least 10 times the predictor degrees of freedom in the model [31]. Therefore, over-fitting of the model may have occurred for some of the subgroup analyses.

People with type 2 diabetes treated with insulin plus concomitant metformin had a reduced risk of death and MACE compared with people treated with insulin monotherapy. There was no statistically significant difference in the risk of cancer between people treated with insulin as monotherapy or in combination with metformin. Further studies are needed to determine the risks and benefits of insulin in type 2 diabetes and the possible benefits associated with the administration of concomitant metformin.
